# Effects of Olive Oil and Its Minor Components on Cardiovascular Diseases, Inflammation, and Gut Microbiota

**DOI:** 10.3390/nu11081826

**Published:** 2019-08-07

**Authors:** Gabriela Marcelino, Priscila Aiko Hiane, Karine de Cássia Freitas, Lidiani Figueiredo Santana, Arnildo Pott, Juliana Rodrigues Donadon, Rita de Cássia Avellaneda Guimarães

**Affiliations:** 1 Graduate Program in Health and Development in the Midwest Region of Brazil, Federal University of Mato Grosso do Sul, Campo Grande 79070-900, Mato Grosso do Sul, Brazil; 2 Laboratory of Botany, Institute of Biosciences, Federal University of Mato Grosso do Sul, Campo Grande 79070-900, Mato Grosso do Sul, Brazil

**Keywords:** Mediterranean diet, *Olea europaea*, monounsaturated fatty acids, phenols, oleuropein, hydroxytyrosol

## Abstract

Olive oil is one of the main ingredients in the Mediterranean diet, being an important ally in disease prevention. Its nutritional composition is comprised of mainly monounsaturated fatty acids, with oleic being the major acid, plus minor components which act as effective antioxidants, such as hydroxytyrosol. Studies have shown that the consumption of olive oil, as well as its isolated components or in synergism, can be a primary and secondary protective factor against the development of cardiovascular diseases since it reduces the concentrations of low-density lipoproteins and increases the concentration of high-density lipoproteins. Furthermore, it exerts an influence on the inflammatory markers, such as interleukin-6 and tumor necrosis factor, which are pro-inflammatory agents in the body. The components present in olive oil are also associated with the promotion of intestinal health since they stimulate a higher biodiversity of beneficial gut bacteria, enhancing their balance. The objective of this review is to present recent data on investigated effects of olive oil and its components on the metabolism, focused on cardiovascular diseases, inflammation, and gut biota.

## 1. Introduction

**Figure 1:**
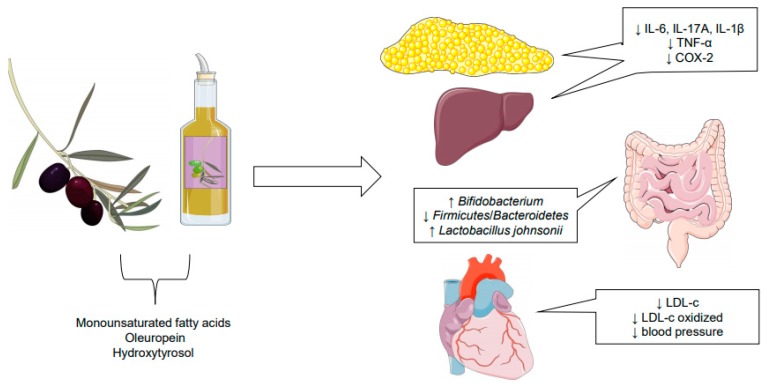
Tumor necrosis factor (TNF-α); interleukin-6 (IL-6); interleukin-17A (IL-17A); interleukin-1β (IL-1β); cyclooxigenase-2 (COX-2); low-density lipoprotein cholesterol (LDL-c).

Regarding current dietary patterns, the Mediterranean diet (MedDiet) is an example of a quality diet that provides protective effects against cardiovascular diseases, inflammation, and cancer. [1] The diet consists mainly of unrefined cereals, fruits, vegetables, beans, fish, nuts, and olive oil, which are responsible for a good health-related quality of life [1]. Olive oil (*Olea europaea*) is considered an excellent source of lipids, being associated with the primary and secondary prevention of cardiovascular disease outcomes, as well as improvement of the lipid profile and insulin sensitivity, increased oxidative stability, improvement of inflammatory markers, and control of arterial pressure [2,3] (Figure 1).

Figure 1 shows the effects of monounsaturated fatty acids, hydroxytyrosol, and oleiropein on cardiovascular diseases, inflammation, and gut microbiota composition.

Such benefits are attributed to its nutritional composition, which is predominantly monounsaturated fatty acids (MUFAs), with oleic acid (C18:1) being the fraction representing 55% to 83%, followed by polyunsaturated fatty acids (PUFAs), representing 4% to 20%, such as linoleic (C18:2) and α-linolenic (C18:3) acids, and saturated fatty acids (SFA), representing 8% to 14%, such as palmitic (C16:0) and stearic (C18:0) acids. It also contains minor compounds, with the phenols oleuropein and hydroxytyrosol standing out nutritionally [1,4,5,6].

Hydroxytyrosol (HT) represents one of the main polyphenol contents of the extra virgin olive oil (EVOO) and has anti-inflammatory and anti-teratogenic activity, improving the lipid profile, reducing oxidative stress, and activating inflammatory cells. [6] It also acts on the expression of peroxisome proliferator-activated receptors (PPAR) γ and α, which reduces the adipocyte size [7]. Furthermore, oleuropein, another antioxidant found in unripe olive fruits and in olive tree leaves, has also been associated with improved inflammatory parameters in various inflammation models, besides consisting of anti-proliferative and anti-tumor properties inducing the apoptosis process of cancer cells, mainly in the colon region [2,5,8]. According to Commission Regulation (EU) No. 432/2012, for polyphenols to be considered as protective against oxidative events, olive oil must contain at least 5 mg of HT per 20 g of olive oil [9].

Furthermore, the consumption of EVOO has been associated with the promotion of gut biota health, promoting the development of a higher biodiversity of intestinal bacteria, with its lipid composition having been shown to have an influence on the ratio between the phyla *Firmicutes/Bacteroidetes* [10]. An increase of the genus C*lostridium XIVa* has also been observed, which is one of the main strict anaerobic groups in the intestine and responsible for the production of butyrate, a short chain fatty acid (SCFA), which has a role in the reduction of total cholesterol and anti-inflammatory activity [10,11].

The objective of this paper is to present a review of recent scientific articles on the metabolic effects that the consumption of olive oil and its components exert on cardiovascular diseases, inflammation, and the gut biota.

## 2. Olive Oil

The MedDiet has been investigated since 1960 and is associated with longevity and well-being with beneficial effects on health, related to a reduction in the development of non-communicable chronic diseases such as cardiovascular disorders and some types of cancer, such as colon and breast cancer [4]. Such benefits are associated with the synergic effects of its components, as the diet is based on the consumption of foods such as fish, nuts, unrefined cereals, fresh fruits, beans, and olive oil [12,13,14].

In relation to its nutritional profile, olive oil is formed of an unsaponifiable fraction which corresponds to its fatty acid profile, being mainly represented by MUFAs. It has a considerable content of minor components (2% of its composition) divided into unsaponifiable and soluble fractions, for which more than 200 compounds have been identified, such as hydrocarbonates, phytesterols, tocopherols, and pigments, among others [1,11].

Commercially, there are different types of olive oil, with EVOO being the most traded worldwide. The United States Department of Agriculture (USDA) has estimated that in 2018/2019, the production of olive oil was 3.09 million metric tons, together with other oils such as coconut, palm, and sunflower, with the European Union being the main global producer [15]. EVOO is obtained by the process of the mechanical pressing of the olive without undergoing any other type of treatment, except the steps of washing, decantation, centrifugation, and filtration. These processes allow the preservation of the minor components, which prevent oxidative events of the oil, besides bringing benefits when consumed [11].

Another commercialized type is refined olive oil, obtained by the mechanical extraction from damaged or inappropriately stored fruits. During the refinement process, loss of part of the phenolic compounds occurs since they do not resist all steps due to their instability, compared with the content found in EVOO. Such losses can reach up to 10 times the total content of phenols compared with EVOO, but, on the other hand, the refinement allows the preservation of fatty acids, which assures the same quality of lipid content found in EVOO [16].

As mentioned before, EVOO has a very good nutritional and sensorial quality, presenting a low acidity index and a value for free fatty acids less than 0.8% [17]. In terms of the lipid content of EVOO, the oleic acid is its major fraction, with content varying from 55–83%, followed by the minor fractions of PUFAs (4–20%), such as linoleic and α-linolenic acids, and those of SFA, such as palmitic and stearic acids. These contents vary according to the degree of ripeness of the olives and local conditions of growth, with fruits from cold places having a higher content of MUFAs [1,4].

The lower content of free fatty acids present in oils is a factor related to a lower risk of developing inflammation. Fatty acids lead to increased cell apoptosis, which is one of the factors related to inflammation, besides generating insulin resistance [18]. Other effects related to EVOO consumption is the control of blood pressure either in normotensive or hypertense individuals. Such effects are associated with the high content of MUFAs, especially oleic acid, which is also responsible for improving other risk factors linked to cardiovascular disorders [19]. The oleic acid also acts on the gastrointestinal system, protecting the intestinal mucosa by means of a reduction of the secretion of chlorhydric acid, preventing ulcers [20].

In addition to presenting antioxidant activity in the body, minor components preserving other components are present, such as vitamin E; additionally, these compounds improve the stability and the nutritional and sensorial quality [1,2,19,21]. Among the antioxidants present in EVOO is HT, which has received attention in the literature of late [6]. This compound exhibits anti-inflammatory and anti-teratogenic activity, improving the lipid profile, and reducing oxidative stress and the activation of inflammatory cells. In addition, it acts on the expression of perixosome proliferators-activated receptors (PPAR) γ and α, which generate reduced adipocyte size [6,7].

Oleuropein, another antioxidant found in unripe olive fruits (14% of dry matter) and in olive tree leaves, has also been associated with the improvement of anti-inflammatory parameters, in different models of inflammation [22]. Additionally, it has presented anti-proliferative and anti-tumor properties induced by the process of the apoptosis of cancer cells [2,5,8]. In one study, animals with colorectal cancer showed a reduction in its size by 64% and 16% when treated with oleuropein at doses of 50 and 100 mg/kg, respectively, and at the highest dose, a reduction of colon inflammation was observed, protecting the epithelium and inhibiting the formation of new tumors there [5]. In olive oil, small amounts of oleuropein are present, and it is more common to observe the derivative forms after hydrolysis such as oleuropein glycoside and aglycon [22].

## 3. Cardiovascular Diseases and Olive Oil

Oxidative stress is a process whereby an imbalance between the oxidant system, which is increased, and the antioxidants, which are reduced, occurs. Such a direct influence in the development of cardiovascular diseases leads to initiation of the atherosclerotic process, as it jeopardizes the endothelial function and increases the oxidation of low-density lipoprotein cholesterol (LDL-c) [11,23]. Cardiovascular disorders are reported as one of the main causes of death worldwide and when associated with risk factors, such as dyslipidemia, arterial hypertension, and inflammation, an increase in the development of the disease and its associated symptoms in metabolism [2]. The avoidable risk factors, such as smoking, sedentarism, and a low-quality diet, correspond to over 90% of the risk of a heart attack, and the improvement of diet quality, such as the increased consumption of whole grains, vegetables, fruits, and good-quality fats, is a factor allied to the correct functioning of adipocytes, having a beneficial impact on cardiovascular complications associated with obesity [8,12].

The endothelial dysfunction causes alterations in the vascular tonus and growth reduction of the heath smooth muscle, besides adherence of the monocytes and other complications associated with the disease. Thereby, the establishment of atherosclerosis occurs, which can lead to the development of associated cardiovascular complications, such as heart attacks and strokes [24]. The intake of EVOO can be utilized as a method of primary prevention for persons who have not developed cardiovascular diseases [2,12].

Effects of primary prevention were reported in a sub-study associated with the *Prevención con Dieta Mediterránea* (PREDIMED), whereby individuals receiving MedDiet plus EVOO showed a reduction in the risk parameters of developing cardiovascular disorders, such as inflammatory cytokine (interleukin-6), the vascular cell adhesion molecule (VCAM) and intercellular adhesion molecule 1 (ICAM-1), and an increase in high-density lipoprotein (HDL-c) levels and diminished LDL-c levels [3]. Endothelial dysfunction results in the accumulation of adhesion molecules which cause vascular lesions. The adhesion molecules are synthesized in endothelial cells and their production is stimulated by the inflammatory cytokines, such as interleukin 1 (IL-1) and tumor necrosis factor (TNF-α). In healthy individuals, a suppression of such adhesion molecules occurs and the reduction in sick individuals indicates less cardiovascular events [25].

Furthermore, the consumption of EVOO can also act beneficially on secondary prevention in sick persons since it exerts control of the LDL-c content and increases the concentration of HDL-c, which facilitates the reverse transport of cholesterol and so slows the illness process, and reduces the risk of future events related to heart problems [2,12,26]. The effects of secondary prevention were observed in another sub-study associated with the PREDIMED (Table 1), whereby the addition of 19 g of EVOO over one year was responsible for improving the functional characteristics of HDL-c in individuals classified as having a high cardiovascular risk. Among the detected effects was the increased cholesterol efflux capacity, which is inversely related to the number of cardiac events, and an increase in the capacity of esterifying the cholesterol and in the liberation of nitric oxide [27].

The oxidation of LDL-c is one of the initial biochemical events in the development of atherosclerosis and coronary disease; when oxidized, it is not recognized by its receptor (LDL-receptor Apo), and is then phagocytosed by macrophages, which leads to the formation of foam cells and induces a cytotoxic response in the endothelial cells, leading to arterial lesion [13,33]. This oxidative process alters the conformation of LDL-c, which is transformed into a toxic molecule, entering the monocytes and causing the development and progression of the atherosclerotic process [1,19].

The phenolic compounds, present in EVOO, act as protective agents against the oxidation of LDL-c, and the oleic fatty acid has the capacity to diminish such oxidation, maintaining the cholesterol efflux and so lowering the circulating LDL-c, thus being more difficult to oxidize and cross the endothelial barrier [1,33]. This result was observed in a study whereby the effects of the MedDiet plus olive oil were evaluated, over one year in 68 people, and an increased resistance of LDL-c to oxidation was observed, as well as diminished alterations caused by the oxidative process and an increase in LDL-c particle size [33].

The phenolic compounds present in EVOO act as antioxidants preventing and reducing events of cardiovascular disease by means of the inhibition of lipid peroxidation caused by free radicals or metals and inhibit the activity of LDL-c, as well as the oxidation of HDL-c. Such phenolic compounds suppress the superoxide reaction and interrupt the oxidation propagation phase [13,23,28]. Contrary to LDL-c, the high content of HDL-c in plasma indicates a lower risk for developing cardiovascular diseases since it transports the cholesterol to the liver, where it is excreted with the bile and then by the feces. In fact, it has been shown that the HDL-c is able to transport the cholesterol from the peripheral tissues to the liver, thus diminishing the circulating content [13,26].

These phenolic compounds are also related to the reduction of mortality associated with cardiovascular diseases, mainly regarding heart attacks and strokes. It has been reported that HT is capable of improving the levels of circulating lipids and repairing oxidative damage associated with cardiovascular diseases [16,34]. In addition, when the polyphenols of EVOO are associated with other compounds, such as the phenolic compounds present in thyme, they have a greater protective effect against the atherosclerotic process, for the diminished oxidated LDL-c, acting in synergy [29].

The protective effect of EVOO against cardiovascular diseases and its risk factors is also associated with its content of MUFAs, especially oleic acid [6,23]. The effect of oleic acid on the heart tissue was observed in animals receiving a high-cholesterol diet plus a lipid source or phenols, demonstrating that the oleic acid improved local inflammation [28]. Such an effect can occur because the MUFAs increase the HDL-c concentrations, reducing the circulating cholesterol, and diminishes the triglyceride level and the trend of HDL-c oxidization, acting on the composition of membranes, benefiting its fluidity and improving the glucose homeostasis [20,28].

The regular consumption of EVOO (25 mL/day) by healthy individuals was found to reduce the LDL-c and total cholesterol, and also control the blood pressure, by means of the negative regulation in the ACE (angiotensin I) and NR1H2 genes, similar to the effects found in individuals receiving 10 mL/day of EVOO plus 3 g/day of fish oil [19,21]. When consumed synergically, EVOO and fish oil diminished the hypercholesterolemia, by means of reducing LDL-c and the ratios of cholesterol total/HDL-c and LDL-c/HDL-c, as EVOO helps in the uptake of omega-3 from fish oil by the cell membranes. Therefore, when consumed together, their benefits are potentialized [21].

Nonetheless, another study observed that the consumption of a high-cholesterol diet (HCD) with EVOO was responsible for increasing the total cholesterol and LDL-c in animals when compared with the group receiving only HCD without the addition of another lipid source. The increase was attributed to the induction of hepatic lipogenic enzymes or a reduced mitochondrial carnitine palmitoyltransferase-1 activity [21]. A similar result was observed in animals receiving HCD with 10% EVOO (with and without the addition of phenols of EVOO) and HCD plus high-oleic sunflower oil (with and without the addition of phenols of EVOO) [28]. The increase of total cholesterol was also noted in individuals receiving olive oil enriched with triterpenes (389 ppm); nevertheless, the content stayed within the limit considered safe against developing cardiovascular diseases [32].

The consumption of EVOO also has an influence on blood pressure, as was observed over 12 weeks of treatment, whereby both systolic and diastolic pressures were lowered when compared with a diet with butter, which has a high content of saturated fatty acids (62.5%) [33]. A decreased diastolic blood pressure was also detected in overweight women consuming 25 mL/day of EVOO for nine weeks, attributed to the anti-hypertensive role of its high concentration of oleic acid [30].

Furthermore, when the olive oil (30 mL/day) was given at different concentrations of phenols and triterpenes (124 and 86, 490 and 86, and 487 and 389 ppm, respectively), a reduction of plasma endothelin-1, which is associated with the development of hypertension and can also be related to inflammation, occurred, but had no effects on sICAM-1 and sVCAM-1 [32]. Nonetheless, other studies have reported that the consumption of EVOO was related to diminished VCAM-1, which acts early at the onset of the atherosclerotic process, indicating once more its protective role in the development of cardiovascular diseases [28].

Other studies found that adherence to the MedDiet also resulted in improvements of the inflammatory process, by means of the reduction of pro-inflammatory cytokines, as well as improving the endothelial function, diminishing atherogenic lipoproteins (LDL-c), and increasing the adiponectin concentration [7]. Adiponectin is formed by adipocytes, found in the blood circulation and has anti-diabetic and anti-inflammatory activity, being a cardioprotective hormone [8]. In obese individuals, its levels are lowered, which causes insulin resistance, arterial hypertension, and the development of atherosclerosis, which are factors predisposing the development of cardiovascular diseases [8]. During the initial steps, the progression of cardiovascular disorders can be influenced by inflammatory reactions, whereby cytokines such as interleukin 6 (IL-6) and TNF-α are induced [23,25].

## 4. Inflammatory Process and Olive Oil

Oxidative stress is also related to the development of inflammation, being generated by the increase of oxygen reactive species (ORS) and nitrogen reactive species (NRS), which are inflammatory agents and which originate from the production of inflammatory cytokines such as IL-6, thus hindering the cell defensive mechanisms’ ability to eliminate ORS and NRS, directly affecting the development and progression of diseases [1,24,35,36]. This process occurs by means of the activation of a signaling cascade after the production of ORS and NRS, which produce and liberate pro-inflammatory cytokines such as IL-6 and activate the Toll-like receptor (TLR)-1, generating new inflammatory cytokines such as IL-8 and TNF-α and thus generating systemic inflammation [18,37].

The adipose tissue liberates inflammatory adipocytokines such as TNF-α and IL-6, and the higher the visceral fat level, the higher the production of these adipocytokines, which triggers the inflammatory process [7]. The increase of adipocytes shortens the blood supply so it is not sufficient for the whole cell, causing hypoxia, which leads these adipocytes to necrosis and infiltration of macrophages into the adipose tissue, causing an increase in the pro-inflammatory markers, such as TNF-α, and in local inflammation [4]. TNF-α is a pro-inflammatory cytokine that causes inflammation via a cascade triggering the serine/threonine kinase, a process which generates the mitogen-activated protein kinases (MAPKs) and transition factors such as nuclear factor (NF)-kβ. These, when occurring together, cause dysfunction in the adipocytes with a reduction of anti-inflammatory cytokines and generate insulin resistance because of inactivation of the receptor in the adipose tissue. The adipocyte, when exposed to TNF-α, triggers the infiltration of monocytes and macrophages, with the subsequent production of IL-6 [4,8,36].

IL-6, another pro-inflammatory interleukin, takes part in the inflammatory regulation process in the responses of the immune system, and its content is increased with the increase of body fat since it can be produced by the adipocytes [4,35]. IL-6 also stimulates the production of C-reactive protein (CRP), which is a marker for the evaluation of systemic inflammation and risk of developing cardiovascular diseases [25,36].

Various reports state that diets with high contents of polyphenols and MUFAs are associated with a reduced production of these cytokines and thus improvement of the oxidative stress and chronic inflammation [11]. In one of the studies, by applying HT and oleic acid on the mouse embryonic fibroblast cell line 3T3-L1, it was possible to note the suppression of TNF-α by interference with the receptor signaling pathways, thus showing anti-inflammatory activity [8]. Furthermore, the intake of HT, at the dose of 50 mg/kg/day by male C58BL/6J mice for eight weeks, decreased the inflammatory markers in the liver (IL-1β and IL-6), as well as reduced the expression of TNF-α, IL-1β, IL-6, Toll-like 4 receptor (TLR-4), and phospho-JNK (p-JNK) also in the liver, and yet the HT promoted a reduction in the liberation of lipopolysaccharides (LPS) in the blood (Table 2) [38].

The effects of EVOO on post-prandial LPS were also observed in individuals with impaired fasting glucose receiving a daily meal containing 10 g EVOO, whereby the circulating LPS stabilized, as well as the oxidation of LDL-c and NADPH oxidase 2 (Nox2), which are markers related to post-prandial oxidative stress [42]. The effects of the consumption of EVOO were also noted in chronic inflammation induced by LPS in piglets receiving a 500 mg/kg EVOO extract with polyphenols and triterpenic acids for 30 days. The supplementation modulated the immune response and impeded the increase of pro-inflammatory cytokines (IL-1β) via an interaction with the signaling cascade of the nuclear factor kappa B (NF-Kβ), indicating once more the protective role of this oil in different experimental models [40].

The synergistic effect of olive oil was again demonstrated when adding phenols (487 ppm) and triterpenes (389 ppm), for three weeks, which were responsible for reducing TNF-α and IL-8 in healthy adults, compared with individuals receiving olive oil with low doses of triterpenes [43]. Such EVOO phenols also have the capacity to reduce NF-kβ and other markers such as malondialdehyde (MDA) [2].

NF-kβ is a primordial transcription factor involved in the regulation process of inflammation in mononuclear cells. A study evaluating post-prandial anti-inflammatory activity showed that EVOO plus phenols resulted in the inhibition of NF-kβ [44]. A similar result was observed in ulcerative colitis cells, where the treatment with oleuropein inhibited the activation of NF-kβ because of the diminished expression of IL-17, associated with the activation of NF-kβ [41]. On the other hand, the administration of 25 mL/kg EVOO with 500 mg/kg EVOO polyphenols resulted in an increase in CRP, indicating an inflammatory process. Such a process can also be observed by the increase in the adhesion of IgA in the gut microbiota, secreted by epithelial cells in the intestinal lumen, which can be utilized as a marker of the inflammatory process since it increases its adhesion to bacteria after changes in gut microbiota [39]. The installation of the inflammatory process may have been caused by the high level of polyphenols since, in excess, they become toxic, acting as pro-oxidants [39].

The inflammatory process is also regulated by the expression of cell adhesion molecules, such as E-selectin, which in this case is increased, and the consumption of EVOO, which has also been shown to be a factor responsible for the diminished E-selectin when compared with the consumption of HCD. Such reduction might be related as much to the presence of oleic fatty acid as to polyphenols, with both having the capacity to reduce the expression of this marker [28].

Other studies report that oleuropein also reduces the production of inflammatory markers (IL-6 and IL-8), at the same time that it increases anti-inflammatory cytokines in colitis models [11]. A similar result was observed in animals with colorectal cancer (azoxymethane model (AOM)/dextran sulfate sodium (DSS)) receiving 50 and 100 mg/kg oleuropein. The animals presented a reduction in the IL-6 and TNF-α content in the colon, as well as in IL-17 at the maximum dose (100 mg/kg) of oleuropein [5]. The effects of this antioxidant were observed in the cyclooxygenase (COX)-2 expression, which is a central mediator of inflammation, mainly related to colon inflammation since it controls the dysfunctions in the intestinal barrier. The application of oleuropein to ulcerative colitis cells was responsible for diminishing the expression of COX-2, caused by the reduction of existing interleukins such as IL-17, as well as diminishing other inflammatory markers such as TNF-α and TL-1β [41].

Such effects were also observed for another group of phenols present in EVOO, the hydroxytyrosol acetate (HTy-Ac), which, when given at the dose of 0.1% in a standard diet, was responsible for improving the clinical signs of ulcerative colitis and regulating the expression of COX-2, inhibiting the activation of NF-kβ and JNK [45]. The reduction of the expression of COX-2 was also observed applying oleuropein at the doses of 50 and 100 mg/kg in animals with colorectal cancer [5]. Indeed, various phenols present in EVOO have protective effects against inflammation, particularly intestinal effects [45].

Some studies have shown that the composition of gut microbiota can be a protective factor against the development of cardiovascular and inflammatory diseases, by means of the reduction of total cholesterol by the action of SCFA, the reduction of available cholesterol for reabsorption, or the formation of protective bioactive metabolites [29].

## 5. Gut Microbiota

The gut microbiota is composed of over 100 trillion cells that have a beneficial role in the maintenance of physiological homeostasis. Its dysregulation, known as dysbiosis, reduces and alters the gut bacteria content, and can modify intestinal permeability, triggering inflammation [11]. It is thus a factor that can lead to metabolic dysfunctions such as obesity, insulin resistance, and the inflammatory process. The ratio of *Firmicutes/Bacteroidetes* is an indicator of dysbiosis [46,47,48].

Balance of the gut microbiota is essential for the maintenance of health in general, whereas an imbalance causes an increase of intestinal permeability, increasing the circulation of LPS and leading to endotoxemia [49]. The LPSs are endotoxins found in gram-negative bacteria related to the activation of the Toll-like receptor 4 (TLR4), which regulates the pro-inflammatory cytokines, and their increase in the blood circulation which can be related to dietetic factors such as the consumption of saturated fats, which stimulate the TLR4 receptors, inducing endotoxemia and inflammation [50].

The variation in gut bacteria content is influenced by genetic, environmental, and dietetic factors, whereby the consumption of EVOO has been associated with the promotion of intestinal health, favoring a higher biodiversity of intestinal bacteria [10]. It has been observed that each type of fatty acid exerts different impacts on the microbiota; however, the isolated mechanisms involved remain unknown. In regard to the groups of bacteria present, the ingestion of lipids has been shown to have an influence on the ratio of the phyla *Firmicutes/Bacteroidetes*, while some polyphenols, such as HT, did not present an influence [10,33,38,47].

The consumption of EVOO has been related to the increase of the genus C*lostridium XIVa*, which is one of the main strict anaerobic groups of the intestine (Table 3). It is responsible for the production of butyrate, which is an SCFA with a role in the reduction of total cholesterol and anti-inflammatory activity and that is one of the main metabolites formed by the gut microbiota [10].

The consumption of EVOO is also related to the multiplication of *Clostridium cocleatum*, as has been previously observed, besides the presence of *Lactobacillus*, which degrades the oleuropein, leading to its fermentation and thus acting as a probiotic [11,31]. Probiotics help in the maintenance of the intestinal barrier by means of various mechanisms, such as increasing the production of mucin; reducing the pro-inflammatory cytokines, such as TNF-α and IL-6; and lowering the concentrations of total cholesterol and LDL-c, among other benefits. Among the bacteria acting as probiotics, *Lactobacillus* and *Bifidobacterium* stand out; both utilize oleuropein as the carbon source, and consequently proliferate, so this antioxidant also acts as a prebiotic [11,48].

The daily intake of HT, at the dose of 50 mg/kg, increases the concentration of *Lactobacillus*, especially *L. johnsonii* [38]. On the other hand, the consumption of EVOO was shown to be associated with the reduction of *Lactobacillus*, especially *L. animalis*, *L. taiwanensis*, and *Lactococcus*, which are microorganisms related to the weight loss of animals receiving EVOO in their diet [31].

As aforementioned, polyphenols influence the microbiota composition, acting as prebiotics; inhibiting pathogenic bacteria growth, such as *E. coli*; and stimulating the probiotic bacteria, such as *Bifidobacterium*. Polyphenols generate a microbial balance since most are not absorbed in the upper gastrointestinal region and reach the colon, where most of these gut bacteria are found [11,51].

Among the bacteria related to the presence of polyphenols, there is the genus *Bacteroides*, which is associated with a protective effect against cardiovascular diseases for diminishing the intestinal permeability and the circulating LPS. The consumption of EVOO for 12 weeks raised the concentrations of *Bacteroides*, especially *B. fragilis* [31,49]. Additionally, the polyphenols of EVOO, when associated with other compounds, such as polyphenols of thyme (500 mg/kg), also favor *Bifidobacterium spp.*, an indicator related to the gut microbiota balance, and *Parascardovia*, indicating the synergic effect of these compounds [29]. The addition of HT also hindered the genera *Proteobacteria*, *Deferribacteres*, and *Rikenella*, besides restoring the gut microbiota depressed by a fat-rich diet and increasing the integrity of the intestinal barrier, which is an effect similar to that of a fecal transplant in animals consuming HT [38].

These polyphenols are also protective against the oxidation of intestinal cells of colorectal adenocarcinoma of the human epithelium, regulating the expression of IL-8 since their properties are capable of eliminating free radicals and metabolites such as sulfates and glucuronides, thus protecting the cells [11]. In Caco-2 cells, extracts of EVOO (varieties Bosana and Neri) provided protection of these cells against tert-butyl hydroperoxide (TBH) and oxysterols, responsible for the increase of EROs which lead the Caco-2 cells to necrosis and apoptosis; the extracts neutralized such effects, preserving the intestinal integrity [52].

## 6. Conclusions

The overall quality of the MedDiet is related to an improved quality of life and the intake of EVOO has indicated in both in vivo animal and human trials that it generates health benefits, such as the prevention of cardiovascular diseases, the improvement of inflammatory parameters and in the composition of the gut microbiota. Such benefits are attributed to the excellent nutritional composition of EVOO, which has a high content of monounsaturated fatty acids (oleic fatty acid) and minor compounds such as polyphenols (oleuropein and hydroxytyrosol). Despite so many positive effects on health, little is known about the mechanisms involved in these processes, especially the isolated actions of the components as well as the possible synergistic effects with other components of the diet. Therefore, further studies in vivo and in vitro are needed to complement the recent findings, in order to understand the mechanisms regarding such components in human and/or animal metabolism, regardless of whether they are healthy or diseased.

## Figures and Tables

**Table 1:** Effects of the consumption of olive oil and its minor components in cardiovascular events and related parameters.

Host	Diet	Effects	References
Metabolic syndrome Adult (*n* = 102)	(1) Control (2) Fish oil (3 g/day) (3) EVOO (10 mL/day) (4) Fish oil + EVOO (3 g/day + 10 mL/day)	(3) ↓ abdominal adiposity (4) ↓ LDL-c, TC/HDL-c and LDL-c/HDL-c	[21]
Male Wistar rats (*n* = 64)	(1) Control (2) HCD (3) HCD + 10% EVOO (4) HCD + 10% sunflower oil (5) HCD + 10% high-oleic sunflower oil (6) HCD + oil-product of EVOO (7) HCD + oil-product of sunflower oil (8) HCD + oil product of high-oleic sunflower.	(3,6,7 and 8)↑ TC and LDL-c	[28]
Male Wistar rats (*n* = 64)	(1) Control (2) HCD (3) HCD + 10% EVOO (4) HCD + 10% sunflower oil (5) HCD + 10% high-oleic sunflower oil (6) HCD + oil-product of EVOO (7) HCD + oil-product of sunflower oil (8) HCD + oil product of high-oleic sunflower oil.	(3, 5, 6 and 8)↑ TC and LDL-c ↓ HDL-c when compared with (2) ↑ MUFA when compared with (1) and (4) (4 and 7) Without alteration in TC and LDL-c ↓ HDL-c when compared with (2) ↑ PUFA	[23]
Male healthy adult (*n* = 18)	(1) 25 mL EVOO (phenol content: 366 mg/kg caffeic acid equivalent) (2) 25 mL de refined EVOO (phenols: 2.7 mg/kg caffeic acid equivalent)	(1) ↓ systolic blood pressure ↓ LDL-c and TV. Negative regulation of the genes ACE and NR1H2 (2) ↑ diastolic blood pressure	[19]
Hypercholesterolemic adult (*n* = 12)	(1) EVOO (2) EVOO + 500 mg/kg EVOO polyphenols (3) EVOO + 500 mg/kg polyphenols of EVOO and of thyme (1:1)	(3) ↓ oxidated LDL-c	[29]
Adult women with excess body fat (*n* = 41)	(1) High-fat breakfast with 25 mL of soybean oil (2) High-fat breakfast with 25 mL of EVOO	(2) ↓ body fat and diastolic blood pressure	[30]
Male Swiss Webster mice (*n* = 26)	(1) Standard diet (2) Standard diet + butter (3) Standard diet + EVOO	(3) Control of blood pressure and ↓ triglycerides	[31]
Healthy adult (*n* = 51)	(1) Olive oil (124 ppm phenols and 86 ppm triterpene) (2) Olive oil (490 ppm phenols and 86 ppm triterpene) (3) Olive oil (487 ppm phenols and 389 ppm triterpeno)	(1,2 and 3) ↓ endothelin-1 plasma (1) ↑ fasting plasma triacylglycerol and ↓ systolic blood pressure (2) ↑ fasting plasma triacylglycerol (3) ↑ TC and ↑ systolic blood pressure	[32]
Adults with risk of cardiovascular diseases (*n* = 210)	(1) MedDiet + olive oil (2) MedDiet + nuts (3) Low-fat diet	(1) ↑ resistance of LDL-c to oxidize, ↓ changes caused by oxidation of LDL-c and ↑ LDL-c cell size	[27]

Abbreviations: ↑: increase; ↓: decrease; extra virgin olive oil (EVOO); low-density lipoprotein (LDL); total cholesterol (TC); high-density lipoprotein (HDL); high-cholesterol diet (HCD); monounsaturated fatty acids (MUFA); polyunsaturated fatty acids (PUFA); Mediterranean diet (MedDiet).

**Table 2:** Effects of the consumption of olive oil and its minor components on inflammation and their related markers.

Host	Diet	Effects	References
Male Wistar albino rats (*n* = 64)	(1) Control (2) HCD (3) HCD + 10% EVOO (4) HCD + oil-product of EVOO (5) HCD + 10% sunflower oil (6) HCD + 10% sunflower oil + oil-product of EVOO (7) HCD + 10% high-oleic sunflower oil (8) HCD + 10% high-oleic sunflower oil + oil-product of EVOO	(3, 4, 7 and 8) ↓ E-selectin (3 and 4) ↓ VCAM-1	[28]
Hypercholesterolemic adult (*n* = 33)	(1) EVOO (2) EVOO + 500 mg/kg polyphenols of EVOO (3) EVOO + 500 mg/kg polyphenols of EVOO and of thyme (1:1)	(2) ↑ CRP and IgA	[39]
Male Wistar rats (*n* = 64)	(1) Control (2) HCD (3) HCD + 10% EVOO (4) HCD + 10% sunflower oil (5) HCD + 10% high-oleic sunflower oil (6) HCD + oil-product of EVOO (7) HCD + oil-product of sunflower oil (8) HCD + oil product of high-oleic sunflower oil	(2) Higher content of TNF-α when compared with (1) (3) ↓ IL-6 TNF-α when compared with (2)	[23]
Female C57BL/6 mice	(1) Control (2) Oleuropein (100 mg/kg) (3) DSS (3 cycles) (4) AOM (1 dose) (5) AOM or DSS (6) AOM or DSS + 5-aminosalicylic acid (75 mg/kg) (7) AOM or DSS + oleuropein (50 mg/kg) (8) AOM or DSS + oleuropein (100 mg/kg)	(3,4 and 5) ↑ IL-17A (7 and 8) ↓ IL-6, IFN-γ, TNF-α (7 and 8) ↓ incidence of colonic neoplasias and inhibited the formation of new tumors.	[5]
Male piglets with subclinical chronic inflammation (*n* = 31)	(1) Negative control (2) Positive control (LPS *E. coli*) (3) Extract olive oil (500 mg/kg + polyphenols and triterpenic acids)	(3) Impeded increased of IL-1β and improved the intestinal integrity	[40]
Cells of patients with ulcerative colitis (*n* = 14)	(1) Cell control (2) Cell stimulated with LPS *E. coli* (3) Cell stimulated with LPS *E. coli* + 3 mM oleuropein	(2) ↑ IL-17 (3) ↓ COX-2, IL-17 and infiltration of leukocytes (3) inhibited activation NF-kβ (3) ↓ production of TNF-α and IL-1β	[41]
Male C57BL/6J mice (*n* = 28)	(1) Control (2) HFD (3) HT diet (50 mg/kg/day) (4) Fecal transplant of the group 3	(2) ↑ LPS and TLR-4, TNF-α, IL-1β, IL-6 and p-JNK (3,4) ↓ LPS, IL-1β and IL-6 ↓ expression TLR-4, TNF-α, IL-1β, IL-6, p-JNK	[38]
Impaired fasting glucose adults (*n* = 30)	(1) Control (2) Lunch with 10 g EVOO	(2) Stabilization LPS, ↓ oxidated LDL-c and ↓ Nox2	[42]
Healthy adult (*n* = 51)	(1) Olive oil (490 ppm phenols and 86 ppm triterpenes) (2) Olive oil (487 ppm phenols and 389 ppm triterpenes) (3) Olive oil (124 ppm phenols and 86 ppm triterpenes)	(2) ↓ TNF-α and IL-8	[43]

Abbreviations: ↑: increase; ↓: decrease; extra virgin olive oil (EVOO); high-cholesterol diet (HCD); vascular cell adhesion molecule-1 (VCAM-1); C-reactive protein (CRP); tumor necrosis factor (TNF-α); interleukin-6 (IL-6); dextran sulfate sodium (DSS); azoxymethane (AOM); interleukin 17A (IL-17A); interferon gamma (IFN-γ); lipopolysaccharides (LPS); interleukin 1β (IL-1β); cyclooxigenase-2 (COX-2); nuclear factor kappa B (NF-kβ); high-fat diet (HFD); hydroxytyrosol (HT); Toll-like receptor-4 (TLR-4); phospho-JNK (p-JNK); NADPH oxidase 2 (Nox2).

**Table 3:** Effects of the consumption of olive oil and its minor compounds on the gut microbiota.

Host	Diet	Effects	References
Hypercholesterolemic adult (*n* = 12)	(1) EVOO (2) EVOO + 500 mg/kg polyphenols of EVOO (3) EVOO + 500 mg/kg polyphenols of EVOO and of thyme (1:1)	(3) ↑ *Bifidobacterium spp.* and ↑ *Parascardovia*.	[19]
Male Swiss Webster mice (*n* = 26)	(1) Standard diet (2) Standard diet + butter (3) Standard diet + EVOO	(3) ↑ *Bacteroides* (*B. fragilis*) and ↑ *Clostridium* ↓ *Lactobacillus* (*L. animalis*, *L. taiwanensis* and *Lactococcus*).	[31]
Male spontaneously hypertensive rats (*n* = 16)	(1) Control (2) EVOO	(2) Higher biodiversity of gut bacteria (*Clostridios XIVa* and *Lactobacillus*).	[10]
Male C57BL/6J mice (*n* = 28)	(1) Control (2) HFD (3) HT diet (50 mg/kg/day) (4) Fecal transplant from group 3	(1) ↓ *Firmicutes*/*Bacteroides* (3 and 4) ↓ *Proteobacteria*, *Deferribacteres* and *Rikenella* (3 and 4) ↑ *Lactobacillus johnsonii* (4) ↓ *Anaeltotruncus sp. G*3	[38]

Abbreviations: ↑: increase; ↓: decrease; extra virgin olive oil (EVOO); high-fat diet (HFD); hydroxytyrosol (HT).
